# Intracellular lactate-mediated induction of estrogen receptor beta (ERβ) in biphasic malignant pleural mesothelioma cells

**DOI:** 10.18632/oncotarget.4486

**Published:** 2015-07-09

**Authors:** Arcangela G. Manente, Giulia Pinton, Sara Zonca, Michele Cilli, Maurizio Rinaldi, Antonio Daga, Stefan Nilsson, Laura Moro

**Affiliations:** ^1^ Department of Pharmaceutical Sciences, University of Piemonte Orientale “A. Avogadro”, 28100, Novara, Italy; ^2^ IRCCS San Martino-IST, 16132, Genova, Italy; ^3^ Karo Bio AB, Novum, S-141 57, Huddinge, Sweden; ^4^ Department of Biosciences and Nutrition, Karolinska Institutet, Novum, S-141 57, Huddinge, Sweden

**Keywords:** biphasic pleural mesothelioma, lactate, estrogen receptor beta, targeted therapy

## Abstract

Biphasic malignant pleural mesothelioma (MPM) is the second most common histotype of MPM. It is histologically characterized by the concomitant presence of epithelioid and sarcomatoid features, the latter associated with worse prognosis.

In this report we describe that silencing of AKT1 in spindle-shaped biphasic MPM cells promotes the shift toward an epithelioid phenotype. Furthermore, AKT1 silencing resulted in decreased expression of the lactate/H+ symporter MCT4 and its chaperone CD147/Basigin, and in the induction of estrogen receptor β (ERβ) expression. We provide evidence that ERβ expression is induced by increased intracellular lactate concentration. Spheroid culturing and tumor growth of ERβ negative biphasic MPM in nude mice resulted in the induction of ERβ expression and response to the selective agonist KB9520. In both models, the treatment with the ERβ agonist results in reduced cell proliferation, decreased expression of MCT4 and CD147/Basigin and increased acetylation and inactivation of AKT1. Collectively, in response to metabolic changes, ERβ expression is induced and exerts an anti-tumor effect through selective agonist activation. The possibility to reverse the more aggressive biphasic mesothelioma histotype by targeting ERβ with a selective agonist could represent a new effective treatment strategy.

## INTRODUCTION

Malignant pleural mesothelioma (MPM) is a highly aggressive cancer associated with asbestos exposure [1–3]. More than 120 million people are estimated to be exposed to asbestos worldwide and the incidence rate is on average 0.1–3.3 per 100,000 individuals, with locally higher incidence rates (5.8 per 100,000) [4–6].

MPM is divided into three histological categories including epithelioid, biphasic and sarcomatoid.

Biphasic tumors are a mix of cube-like epithelial cells and spindle-shaped sarcomatoid cells [7]. These two cell types typically reside in separate areas of the tumor, but they can also develop close together. How well a biphasic tumor responds to treatment depends on the ratio of epithelial to sarcomatoid cells. A tumor composed mostly of epithelioid cells grows more slowly and responds better to treatment [8, 9].

MPM is highly resistant to therapy and the preferred treatments are surgery (at early stages) and/or combinatorial treatments with radiotherapy and chemotherapy [10]. Conventional therapy with cisplatin and pemetrexed allows only palliation for the majority of patients and the average survival time after diagnosis remains poor [11–13]. There is, therefore, a pressing need for advancement in the understanding of the disease pathobiology and progression, and in the development of more effective therapies.

The PI3K/AKT signaling pathway is aberrantly active and has an important biologic impact in MPM progression and chemo-resistance [14–17]. Once activated, PI3K and its downstream effector AKT1 not only provide strong growth and survival signals to tumor cells, but also exert profound effects on their metabolism [18].

In addition to intracellular signaling pathways, the abnormal tumor microenvironment, characterized by limited nutrient and oxygen supply, have a major role in determining the metabolic phenotype of tumor cells. The importance of metabolic restriction in cancer has often been masked, due to the use of cell culture conditions in which both oxygen and nutrients are always in excess. In solid tumors, functionally abnormal vasculature combined with altered tumor cell metabolism, creates spatial and temporal heterogeneity in oxygenation, acidity and concentration of metabolites. These extreme conditions induce a collection of cellular stress responses that further modulate the metabolic phenotype of tumor cells and influence tumor progression [18].

It is well known that tumor hypoxia in some cases promotes malignant progression, invasion and acquisition of resistance to chemotherapy and radiation whereas in other cases it inhibits cell proliferation and induces differentiation, apoptosis or necrosis [19].

Under hypoxia, cancer cells develop an adaptive metabolic response, activating glucose uptake and glycolysis to produce pyruvate, which is subsequently converted into lactate.

Monocarboxylate transporters (MCTs), also called the solute carrier family 16 (*SLC16*), especially MCT1 (*SLC16A1*) and MCT4 (*SLC16A3*) and their chaperone Basigin (also named CD147 and EMMPRIN) are crucial in regulating lactate entry or export across the plasma membrane and to decrease high intracellular lactate levels resultant from glycolytic activity [20]. There are controversial data on MCTs regulation by hypoxia, some evidence points at hypoxia-mediated increase of MCT4, but not MCT1, whereas other data indicate that both MCT isoforms are regulated by hypoxia [21, 22]. Marked increases in the levels of MCT1 and/or MCT4 are hallmarks of several human malignancies, and high levels of these transporters connote poor outcome [23–26].

Low oxygen tension modulates cancer cell metabolism through stabilization of hypoxia-inducible factor (HIF)-α subunits (HIF-1α, HIF-2α, and HIF-3α) that in turn translocate to the nucleus where they heterodimerize with the constitutive HIF1-β, also referred to as aryl hydrocarbon receptor nuclear translocator (ARNT), and the recruitment of cofactors [27]. The recruitment of ARNT is critical for the HIF complex to bind to hypoxia-responsive elements (HRE) on target genes and activation of their transcription. ARNT has also been claimed to directly interact with estrogen receptors (ERs) and has been shown to co-activate ER-dependent gene expression [28]. It has been reported that ERβ inhibits HIF-1α-mediated transcription by targeting ARNT, via ubiquitination processes, which may in part account for the tumor suppressive function of ERβ [29, 30].

Our group has recently demonstrated that ERβ exerts a key role as a tumor-suppressor gene in MPM [31]. We reported that MPM cell proliferation and tumor growth can be effectively suppressed by selective agonist activation of ERβ and that the growth inhibitory efficacy is related to the level of ERβ expressed. Furthermore, we recently described that activation of ERβ with the selective agonist KB9520 increased the sensitivity of MPM tumors to the cisplatin/pemetrexed combination, providing evidence that drugs that selectively activate ERβ might prove promising in the development of novel, targeted therapies for the clinical management of human MPM [32].

In the present study, we describe a role for lactate, accumulated in hypoxic conditions, in the modulation of ERβ expression in ERβ-null cells originally derived from a patient with biphasic MPM. Moreover, we explore the efficacy of the selective ERβ agonist KB9520 to suppress biphasic malignant cell proliferation *in vitro* and tumor growth *in vivo*.

## RESULTS

### AKT1 modulation in MPM cells affects ERβ expression and cell metabolism

**Figure 1:**
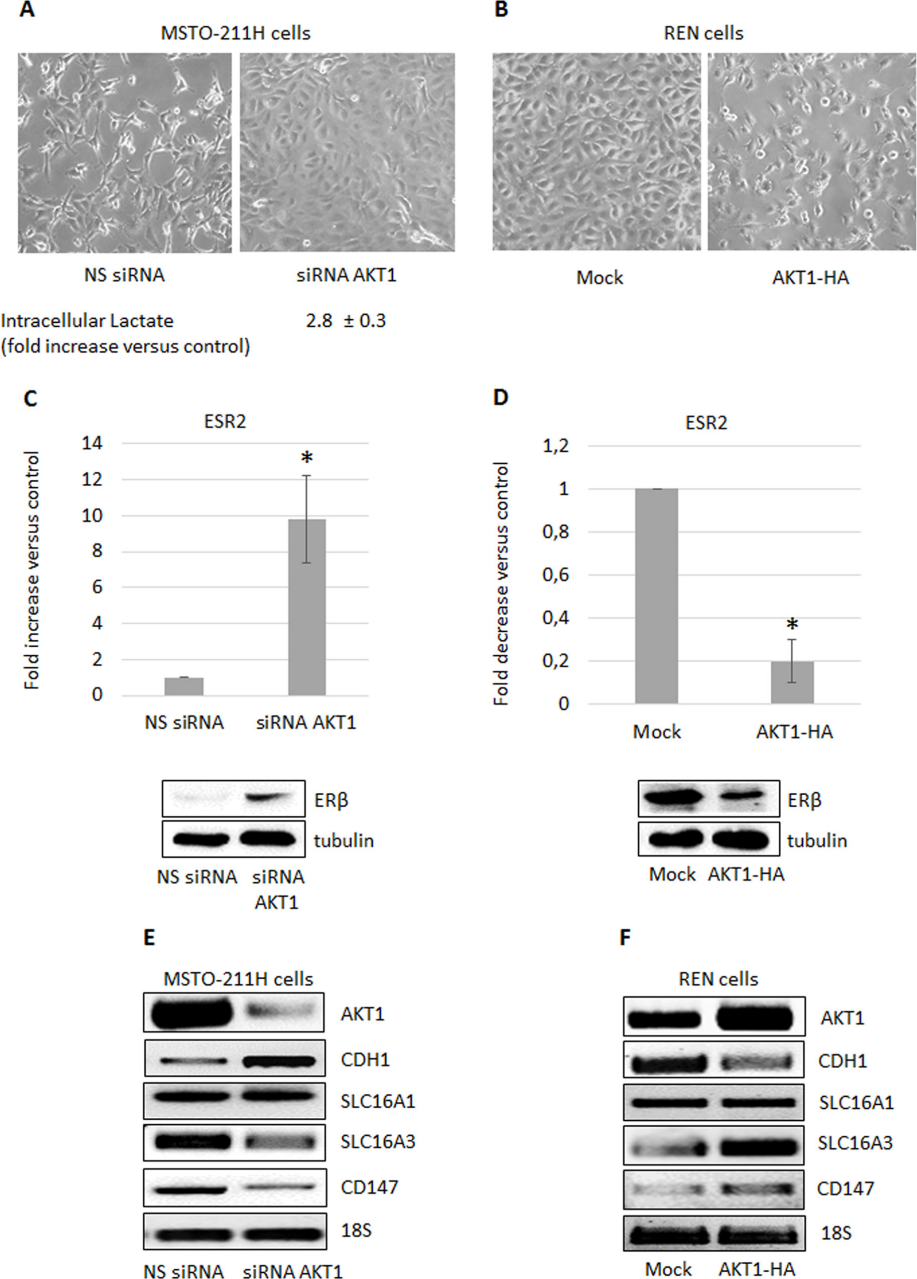
AKT1 modulation in MPM cells affects cell metabolism and ERβ expression **A.** Phase contrast images (200X magnification) of MSTO-211H cells transfected with non-specific control siRNA (NS siRNA) or AKT1 specific siRNA (siRNA AKT1) and **B.** of mock- or AKT1-HA transfected REN cells. Below the MSTO-211H images is reported the mean ± s.d. increase of intracellular lactate compared to control. **C.** ERβ mRNA expression evaluated by real-time PCR in control (NS siRNA) or AKT1-silenced (siRNA AKT1) MSTO-211H cells and **D.** in mock- or AKT1-HA transfected REN cells. Each graph is representative of three independent experiments. Each bar represents mean ± s.d. *p ≤ 0.05. Below each graph is reported a representative Western blot image of ERβ expression. Tubulin is included as a loading control. **E. F.** Representative RT-PCR analyses of *AKT1*, *CDH1*, *SLC16A1* and *3*, and *CD147* in transfected MSTO-211H and REN cells compared to their controls. 18S rRNA was used as housekeeping gene.

We recently published that MPM derived cell lines express AKT1 and AKT3 but not AKT2 [17]. In this study we investigated the AKT1 isoform-specific functions in two MPM derived cell lines. We transiently silenced AKT1 expression in MSTO-211H (high expresser) and transiently overexpressed it in REN (low expresser) cells. AKT1 silencing in MSTO-211H cells resulted in an increase in intracellular lactate content and a morphological shift toward an epithelioid phenotype compared to cells transfected with non-specific siRNAs (Figure 1A). In contrast, overexpressing AKT1 in REN cells induced a more spindle-like phenotype (Figure 1B). As shown in Figure 1C, ERβ was significantly induced both at mRNA and protein levels in AKT1 silenced MSTO-211H cells, whereas ERβ expression was significantly inhibited by AKT1 overexpression in REN cells (Figure 1D). We recently described the metabolic impact of ERβ expression and agonist activation in MPM cells [33, 34]. In present study, we observed that also AKT1 plays an important role in the modulation of different genes coding for proteins involved in metabolic pathways. In particular, the expression of the *SLC16A3* and *CD147* genes was significantly down-regulated in AKT1 silenced MSTO-211H cells and significantly up-regulated in REN cells overexpressing AKT1 (Figure 1E, 1F). No significant change in *SLC16A1* expression was observed (Figure 1E, 1F). Moreover, consistent with morphological changes, we observed the modulation of *CDH1* and *VIM* expression (Figure 1E, and Supplementary Figure S1).

### Increased intracellular lactate induces ERβ expression

**Figure 2:**
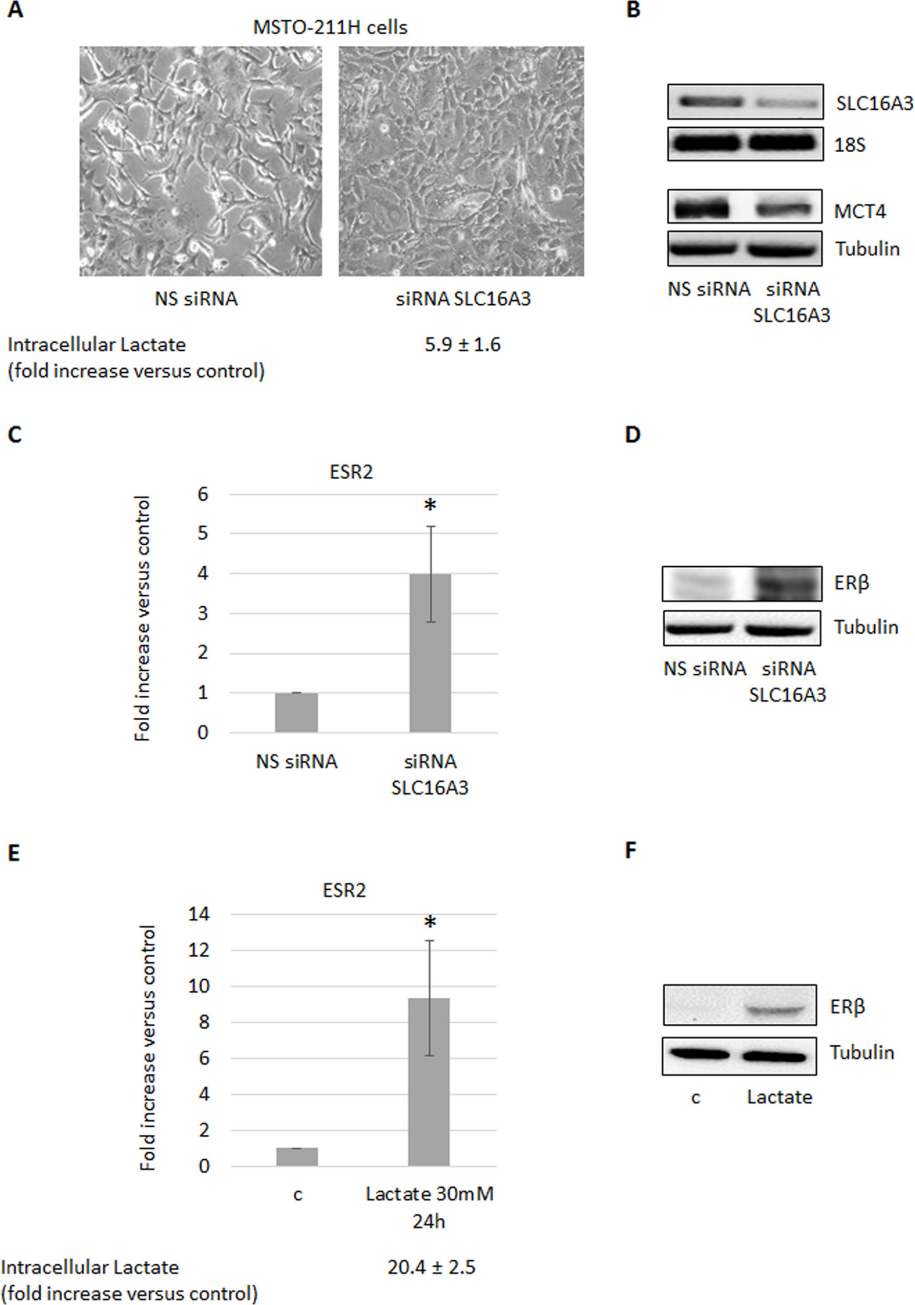
Increased intracellular lactate induces ERβ expression **A.** Phase contrast images (200× magnification) of MSTO-211H cells transfected with non-specific control siRNA (NS siRNA) or *SLC16A3* specific siRNA (siRNA SLC16A3). Below images is reported the mean ± s.d. increase of intracellular lactate compared to control. **B.** RT-PCR and Western blot analyses that confirm *SLC16A3* silencing. 18S rRNA and tubulin were used as loading controls. **C.** ERβ mRNA expression levels evaluated by real-time PCR and **D.** Western blot analyses in control (NS siRNA) or *SLC16A3* silenced (siRNA SLC16A3) MSTO-211H cells. **E.** ERβ mRNA expression evaluated by real-time PCR and **F.** Western blot in MSTO-211H cells treated 24 hours with 30 mM lactate. Below the graph is reported the mean ± s.d. increase of intracellular lactate compared to control. Each graph is representative of three independent experiments. Each bar represents mean ± s.d. *p ≤ 0.05.

**Figure 3:**
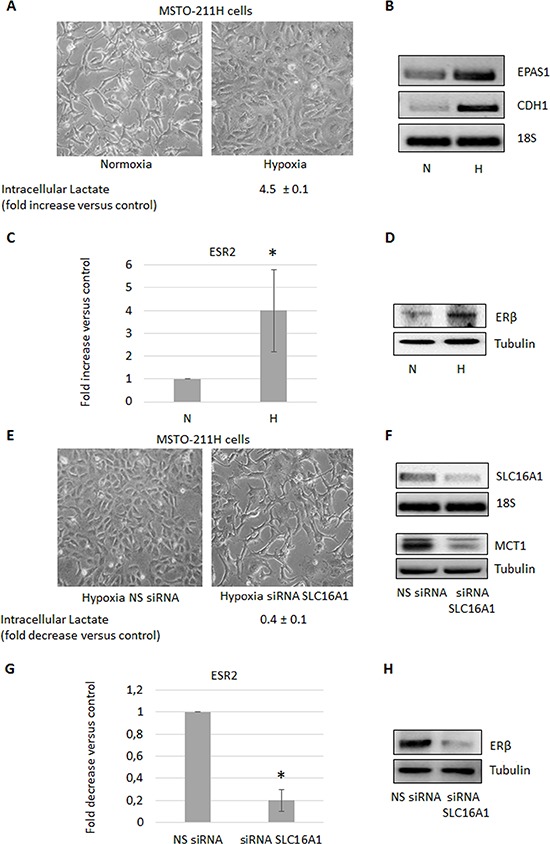
Hypoxia induces the increase of intracellular lactate and ERβ expression **A.** Phase contrast images (200× magnification) of MSTO-211H cells cultured 48 hours in normoxic or hypoxic conditions. Below images is reported the mean ± s.d. increase of intracellular lactate compared to control. **B.** Reverse transcription-polymerase chain reaction (RT-PCR) analyses of *EPAS1* and *CDH1* expressed by MSTO-211H cells cultured 48 hours in normoxia (N) or hypoxia (H). **C.** ERβ mRNA expression evaluated by real-time PCR and **D.** Western blot analyses in MSTO-211H cells cultured 48 hours in normoxic or hypoxic conditions. 18S rRNA and tubulin, respectively, were used as loading controls. **E.** Phase contrast images (200X magnification) of MSTO-211H cells transfected with non-specific control siRNA (NS siRNA) or *SLC16A1* specific siRNA (siRNA SLC16A1) in hypoxic culture conditions. Below images is reported the mean ± s.d. intracellular lactate levels compared to control. **F.** RT-PCR and Western blot analyses that confirm *SLC16A1* silencing. 18S rRNA and tubulin, respectively were used as controls. **G.** ERβ mRNA expression evaluated by real-time PCR and **H.** Western blot analyses in control siRNA (NS siRNA) or *SLC16A1* silenced (siRNA SLC16A1) MSTO-211H cells cultured 48 hours in hypoxia. Each graph is representative of three independent experiments. Each bar represents mean ± s.d. *p ≤ 0.05.

To examine if the observed increase in intracellular lactate could be involved in the induction of ERβ expression, we silenced the *SLC16A3* gene, coding for the lactate exporter MCT4, in the ERβ-null MSTO-211H cells cultivated at normoxic conditions. As expected, the functional consequence of *SLC16A3* silencing was an increase (more than five times) in the content of intracellular lactate (Figure 2A, 2B). As shown in Figure 2C and 2D, this resulted, in turn, in the induction of ERβ expression at both the mRNA and protein levels. In addition, silencing of *SLC16A3* in normoxic MSTO-211H cells induced similar epithelioid-like phenotype as AKT1 silencing (see Figure 2A and 1A). To further explore the role of lactate in the stimulation of ERβ expression, MSTO-211H cells were treated with 30 mM lactate, which in parallel to an increase in intracellular lactate also lead to the induction of ERβ expression (Figure 2E, 2F). Moreover, MSTO-211H cells cultured under hypoxic conditions for 48 hours showed increased intracellular levels of lactate, which was associated with an epithelioid phenotype (Figure 3A) and increased expression of the HIF-2 (EPAS1), E-Cadherin (CDH1) (Figure 3B) and ERβ (ESR2) coding genes (Figure 3C, 3D). Further support for the role of lactate in the modulation of ERβ expression, the *SLC16A1* gene, coding for the lactate importer MCT1, was silenced in MSTO-211H cells cultured at hypoxic conditions. *SLC16A1* silencing resulted in changed cellular phenotype from epithelioid to spindle-like (Figure 3E, 3F), decreased intracellular lactate content (Figure 3E), and decreased ERβ expression (Figure 3G, 3H).

### MSTO-211H cells cultured at hypoxic conditions or as spheroids acquire sensitivity to the ERβ selective agonist KB9520

**Figure 4:**
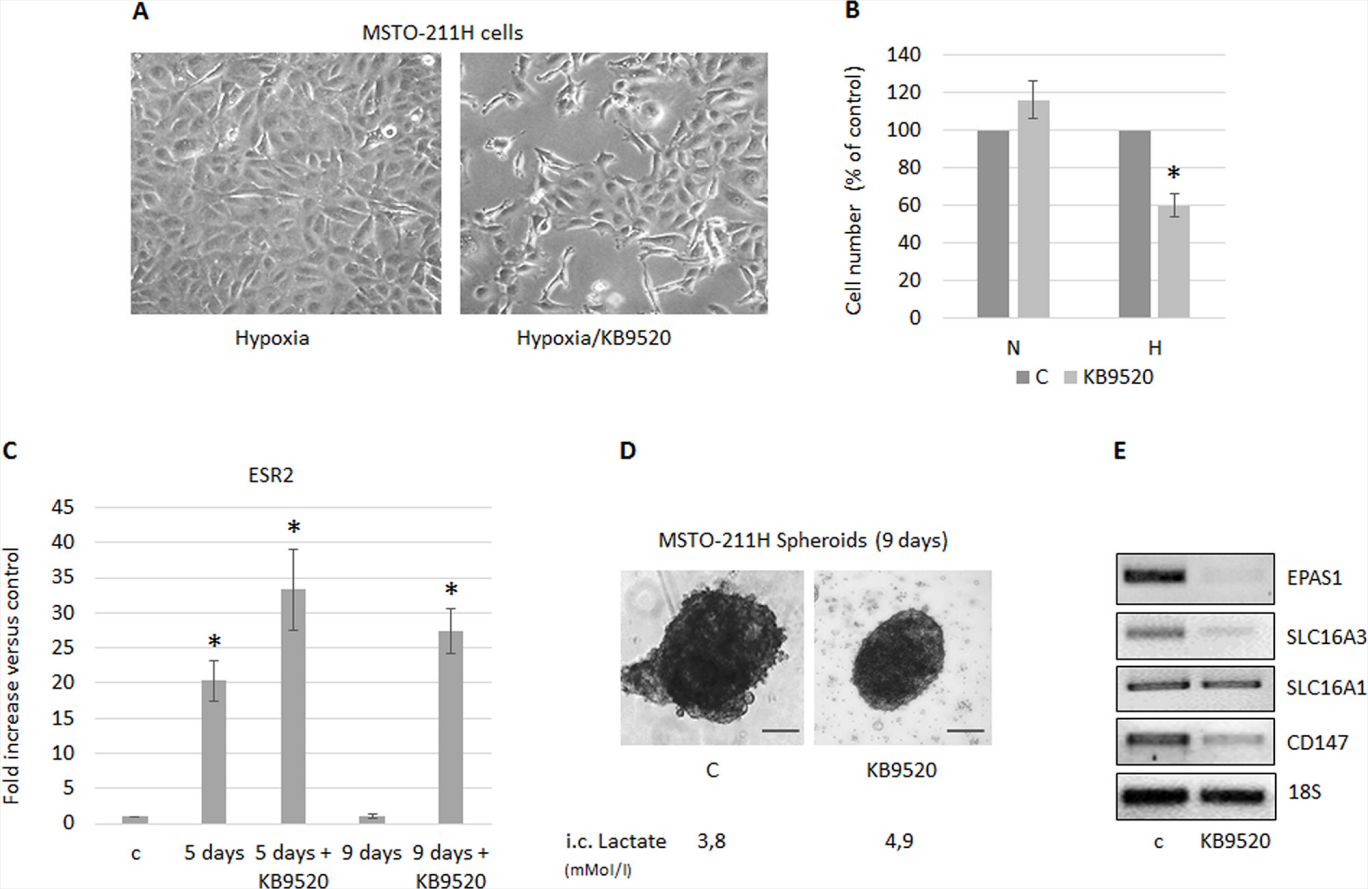
MSTO-211H cells cultured in hypoxic conditions or as spheroids acquire sensitivity to KB9520 **A.** Phase contrast images (200× magnification) of MSTO-211H cells cultured 48 hours in hypoxic conditions ± 10 nM KB9520. **B.** Percentage of growth inhibition in MSTO-211H cells treated with 10 nM KB9520 in normoxic (N) or hypoxic (H) conditions for 48 hours. **C.** ERβ mRNA expression levels evaluated by real-time PCR in MSTO-211H spheroids (pools of 5) grown for 5 or 9 days ± 10 nM KB9520. **D.** Phase contrast images (200× magnification) of MSTO-211H spheroids grown for 9 days ± 10 nM KB9520. Bar equals 100 μM. **E.** Representative RT-PCR analyses of *EPAS1*, *SLC16A1 - 3* and *CD147* expressed by MSTO-211H spheroids (pools of 5) grown for 9 days ± 10 nM KB9520. 18S rRNA was used as housekeeping gene. Each graph is representative of three independent experiments. Each bar represents mean ± s.d. *p ≤ 0.05.

MSTO-211H cells cultured at normoxia do not express ERβ and subsequently, and as previously reported, do not respond to KB9520 [34]. However, since MSTO-211H cells do express ERβ when cultured in hypoxic conditions, we tested their response to the selective ERβ agonist KB9520. As expected and in agreement with previously published data [32], KB9520 significantly reduced the cell growth of MSTO-211H cells cultivated in hypoxic conditions (Figure 4A, 4B). Similar sensitivity and growth inhibitory effect of KB9520 was also observed in MSTO-211H cells, silenced for *SLC16A3* gene expression in normoxia (data not shown). ERβ expression and response to the selective agonist KB9520 was also evaluated in packed MSTO-211H spheroids. Multicellular spheroids, differently from monolayer cultures, represent a useful *in vitro* model that mimics spatial oxygen, glucose, and lactate gradients present in *in vivo* under-vascularized tumors. Interestingly, by RT-PCR and Western blot analysis, we observed a transient induction of ERβ expression between the third and fifth day of spheroid growth, that was sustained until the ninth day only in those treated with KB9520 (Figure 4C). Furthermore, treatment of spheroids with KB9520 for nine days resulted in a significant growth inhibition and lactate accumulation (Figure 4D). By RT-PCR, we documented that at the end of the KB9520 treatment the expression of the *EPAS1*, *SLC16A3*, *CD147* genes were down-regulated, whereas *SLC16A1* expression remained unaffected (Figure 4E).

### The ERβ selective agonist KB9520 promotes ERβ expression and reduces MSTO-211H tumor growth *in vivo*

**Figure 5:**
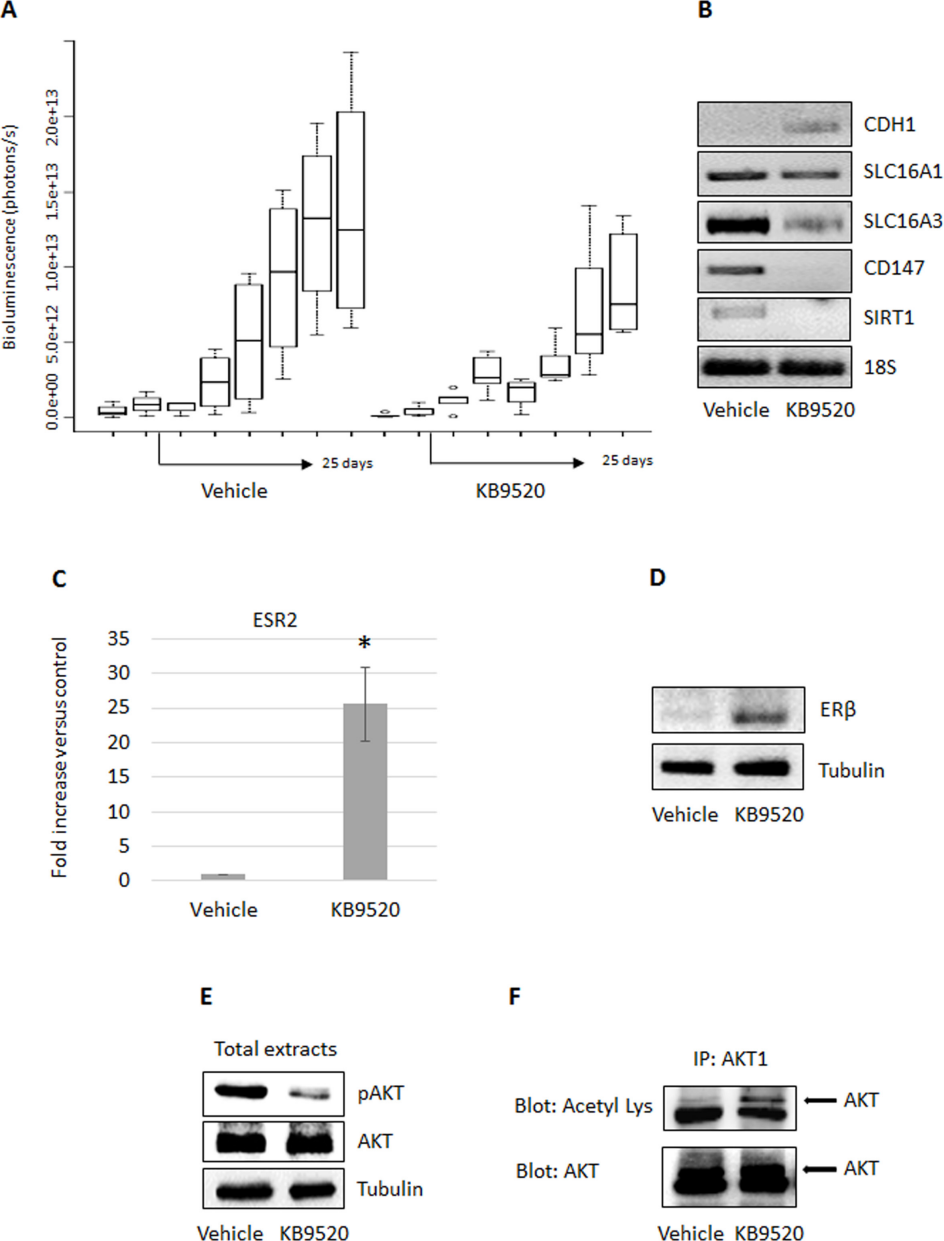
KB9520 sustains ERβ expression and reduces tumor growth *in vivo* **A.** Box plots showing *in vivo* mean ± s.d tumor growth evaluated in MSTO-211H/Luc cells implanted in CD1 nude mice (8 mice/group), treated with vehicle or KB9520 (10 mg/kg body weight/day) for 25 days. **B.** Representative RT-PCR analyses of *CDH1*, *SLC16A1* - *3*, *CD147* and *SIRT1* in tumors recovered from CD1 nude mice (pools of three), treated with vehicle or KB9520 (10 mg/kg body weight/day) for 25 days. **C.** ERβ expression evaluated by real-time PCR and **D.** Western blot in tumors recovered from CD1 nude mice (pools of three), treated with vehicle or KB9520 (10 mg/kg body weight/day) for 25 days. The graph is representative of three independent experiments. Each bar represents mean ± s.d. *p ≤ 0.05. **E.** Representative Western blot analysis of phosphorylated and total AKT in tumor tissues from vehicle and KB9520 treated mice. **F.** Representative immunoprecipitation analysis of acetylated AKT1 in tumor tissues from vehicle and KB9520 treated mice.

Based on the *in vitro* results, we decided to test if ERβ expression was induced and if the selective ERβ agonist could affect MSTO-211H tumor growth in an *in vivo* mouse model. Six weeks old CD1 nude male mice were inoculated intra peritoneum with 1 × 106 MSTO-211H cells (2 groups, 8 animals per group). Prior to inoculation, the MSTO-211H cells were transduced with a lentivirus vector carrying the luciferase gene, to allow imaging in live mice. Treatment of the animals was initiated 15 days after cell inoculation when tumor take-rate in the peritoneal cavity was 100% in the two animal groups. The ERβ-selective agonist KB9520 was administered on day 15 through 40 by subcutaneous injection at 10 mg/kg/day. Untreated animals were subcutaneously dosed with empty vehicle. Tumor size was assessed by IVIS imaging performed every 4–5 days. Starting from 10 days of treatment, we observed a reduction in tumor growth in the group treated with KB9520 as compared to the vehicle treated animals, an effect that was maintained until the end of the experiment (Figure 5A). Treatment with KB9520 was not toxic as assessed by monitoring changes of mice body weights during drug administration (data not shown). 40 days after MPM cell inoculation, all animals were sacrificed and tumors were dissected and immediately frozen. By RT-PCR of tumor samples, we documented that KB9520 treatment decreased the expression of the *SLC16A3* and *CD147* genes and increased the expression of the *CDH1* gene. The expression of the *SLC16A1* gene remained, as in earlier experiments, unaffected (Figure 5B). By RT-PCR and Western blot analysis, we also observed that tumor samples from mice treated with KB9520 had increased ERβ expression both at mRNA and protein levels compared to vehicle treated animals (Figure 5C, 5D). Moreover, the level of phosphorylated AKT was significantly lower in tumor cells from KB9520 treated mice compared to vehicle animals (Figure 5E). In contrast, there was an increase in AKT1 acetylation (Figure 5F). As recently described, acetylated AKT, due to SIRT1 inactivation, prevents its correct localization and phosphorylation [35]. We therefore decided to analyze the expression of *SIRT1* in MSTO-211H tumors recovered from mice; *SIRT1* expression was reduced in tumors from KB9520 treated mice (Figure 5B). Similar AKT1 post-translational modifications and *SIRT1* down-regulation were also observed in KB9520 treated MSTO-211H cells cultivated in hypoxic conditions (Supplementary Figure S2).

## DISCUSSION

The serine-threonine kinases family AKT (also known as PKB) consists of three highly homologous isoforms: AKT1 (PKBα), AKT2 (PKBβ) and AKT3 (PKBγ), encoded by different genes [36]. Findings from AKT isoform-specific knockout mice suggest that the functions of the different AKT kinases are not completely overlapping and that isoform-specific signaling contributes to the diversity of AKT activities [37]. There is evidence for hyperactivation of specific AKT isoforms in certain tumors, suggesting that in some cases there is an AKT isoform-specificity to cell transformation [38]. Although AKT is one of the most frequently hyperactivated kinase in human MPM, isoform specificity remains to be explored. We recently published that MPM derived cell lines express AKT1 and AKT3 but not AKT2 [17]. In the present study, we explored the effects of AKT1 modulation in MPM derived cell lines representative of the epithelioid and the biphasic histotype, characterized by different levels of basal AKT1 expression. AKT1 modulation *in vitro* led to marked changes in the cell morphology and, consistent with this, in *CDH1* expression, suggesting a role for the AKT1 isoform in epithelial-mesenchymal transition (EMT) and in the reciprocal mesenchymal-epithelial transition (MET). Interestingly, the observed changes in cell morphology in response to AKT1 modulation resembled those previously reported following ERβ expression modulation in the same cell types [33]. Results presented in this report suggest that AKT1 is involved in the modulation of ERβ expression. In our previous studies, we documented the role of ERβ as tumor suppressor and positive prognostic factor in patients diagnosed with MPM and described that one of the mechanisms by which activated ERβ exerts tumor suppressive function is the alteration of aerobic energy metabolism, impairing mitochondrial respiratory chain complexes and forcing cells to depend on glycolysis [34]. The regulation of metabolism in cancer by the AKT kinases is a very active area of research, but there is a limited understanding about the role of the AKT family members. Here we describe that, in MPM cells, AKT1 modulates the expression of *SLC16A3* and *CD147*, coding for the two proteins MCT4 and Basigin, which are involved in metabolic pathways. MCTs are proteins that facilitate the transmembrane transport of short-chain fatty acids, such as lactate, coupled with a proton. The release of lactate occurs mainly through MCT4, whereas its uptake occurs through MCT1 [39]. Of note, the MCTs (primarily the MCT1 and MCT4) and Basigin are overexpressed in most tumors, including MPM [40]. Basigin has been described to associate with the MCT transporter isoforms MCT1 and MCT4, acting as a chaperone and facilitating their cell surface expression and/or appropriate insertion and location. Targeting MCTs/Basigin complexes was reported to impair the *in vitro* and *in vivo* growth of pancreatic tumor cells, Ras-transformed fibroblasts, colon adenocarcinoma, and Myc-induced human malignancies, suggesting that blocking lactic acid export provides an efficient metabolic therapy to limit tumor cell growth [41]. Lactate was originally thought to be an acidic molecule, which must be exported to prevent deleterious intracellular acidification. Recently, different roles of lactate export/import have been implicated in tumor growth [42]. For example, it has been described that lactate exerts a transcriptional effect acting like a histone deacetylase (HDAC) inhibitor, linking the metabolic state of the cell to gene transcription. Lactate is a relatively weak inhibitor, compared to the established HDAC inhibitors, but the repertoire of genes regulated overlap [43]. Here we provide data that the increase in intracellular lactate content induced ERβ expression in MSTO-211H cells, as evidenced by addition of lactate to the growth medium, or its increase due to knock-down of MCT4 expression or by culturing cells in hypoxic condition. Based on the results following treatment of MSTO-211H cells with the established HDAC inhibitor SAHA (Supplementary Figure S3) we find the role of lactate as HDAC inhibitor to be the most plausible mechanistic explanation behind the increased expression of ERβ in the MSTO-211H cells.

We used the 3-dimensional tumor cell model of spheroids to further explore the role of lactate and/or hypoxia in ERβ modulation. Since the cells in spheroids are not grown in a monolayer, they are exposed to varying degrees of oxygenation and nutrient availability based on their spatial distribution. Moreover, the spheroid system circumvents a major problem of tumor analysis *in vivo*, which is the difficulty of distinguishing between local environmental effects and differences due to cellular heterogeneity; multicellular tumor spheroids mimic the development patterns of *in vivo* avascular tumor nodules in terms of morphology and growth kinetic properties [44]. When we cultured the ERβ negative MSTO-211H cells as spheroids *in vitro*, ERβ was rapidly induced and then lost. We believe that this transient expression of ERβ is best explained by the uptake and rapid conversion of lactate to pyruvate, by the peripheral normoxic cells in metabolic symbiosis with hypoxic cells of the spheroid core, thereby preventing intracellular accumulation of lactate. In contrast, when ERβ was activated by its selective agonist KB9520, its expression was maintained and cell growth significantly inhibited. In spheroids treated with KB9520, *SLC16A3* and *CD147* expression were down-regulated, causing an increase in intracellular lactate that could in part explain the maintenance of ERβ expression.

**Figure 6:**
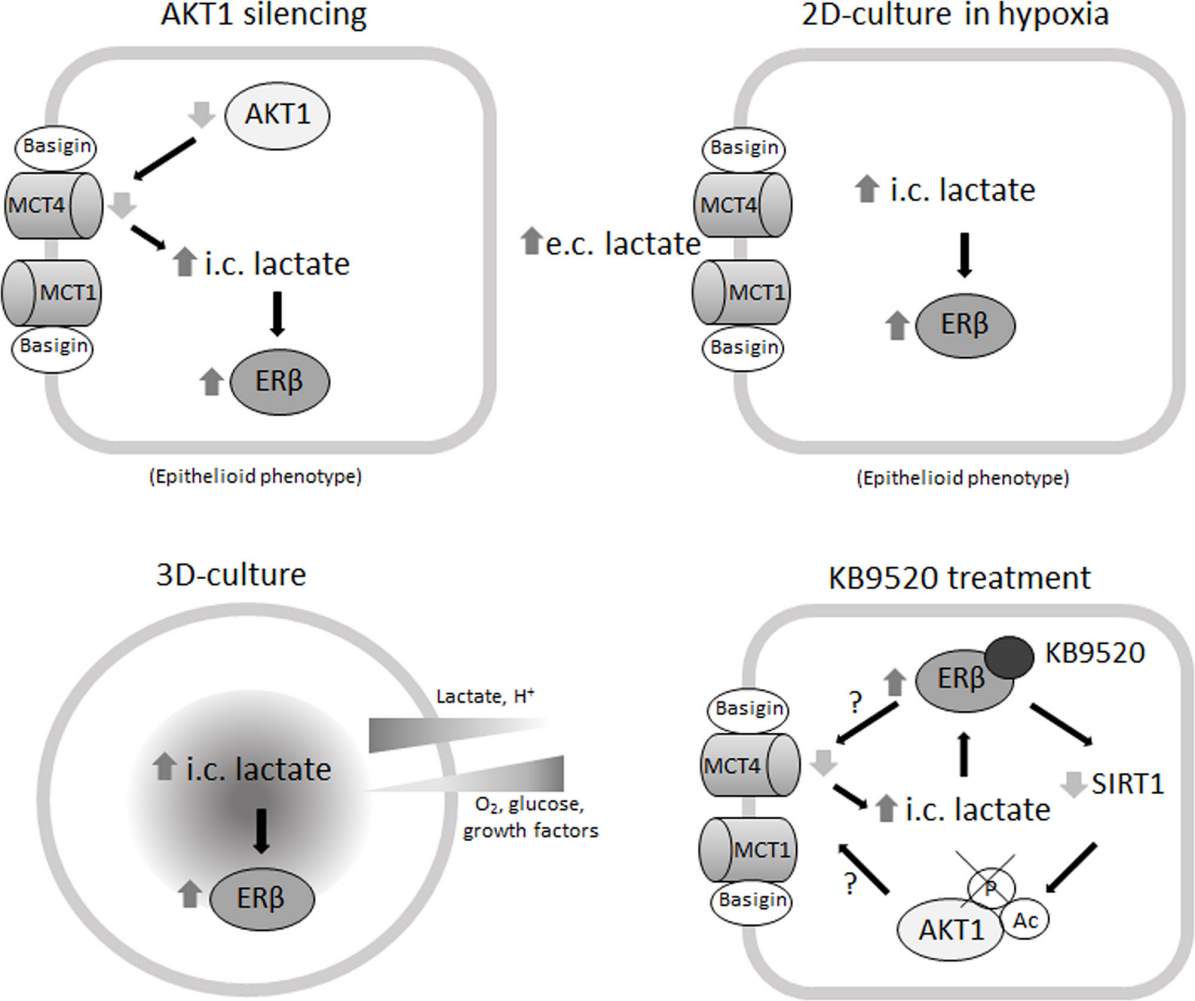
Mechanisms of ERβ induction and KB9520 action in biphasic MPM cells

We also explored ERβ expression and response to KB9520 in an *in vivo* MSTO-211H mouse model. After an initial phase of latency in which tumors transiently expressed ERβ, probably due to changes of environmental conditions, we observed reduced tumor growth in mice treated with KB9520. Similar to observations in spheroids, ERβ expression was maintained and *SLC16A3* and *CD147* gene expression were down-modulated in tumor samples recovered from KB9520 treated mice. Moreover, both *in vitro* and *in vivo* we observed that activated ERβ significantly inhibited AKT phosphorylation/activation, which could represent another mechanism that explains its sustained expression and tumor suppressive activity. A possible explanation for the reduced phosphorylation of AKT is the observed increase in its acetylation. It has been reported that acetylation blocks binding of AKT and PDK1 to PIP(3) [35], thereby preventing its membrane localization and phosphorylation whereas deacetylation by SIRT1 promotes AKT activation. Mice injected with cells expressing a mutated AKT that mimicked a constitutively acetylated form of AKT, developed smaller tumors than those injected with cells expressing wild-type AKT [35]. Consistent with decreased expression of *SIRT1 in vitro* and *in vivo* we observed increased AKT1 acetylation. The reduced activation of AKT, along with the reduction in expression of *SLC16A3* and *CD147*, could be the explanation for the reduced tumor growth. Collectively, the presented data suggest that metabolic changes can promote, via epigenetic modulations, ERβ expression in ER negative cells and that its expression and tumor suppressive function are maintained by its selective ligand activation (Figure 6). The prognosis for patients diagnosed with biphasic mesothelioma is less favorable than those diagnosed with epithelioid mesothelioma and depends on the ratio of sarcomatoid and epithelioid cells in the biphasic tissue. The possibility to reverse the more aggressive biphasic cell phenotype by targeting ERβ with a selective agonist could represent a new strategy to effectively treat this histological subtype of mesothelioma.

## MATERIALS AND METHODS

### Reagents and antibodies

The monoclonal antibodies specific for α-Tubulin, AKT1 and Acetylated-Lysine and the polyclonal antibodies specific for ERβ, phospho-AKT (pSer473), AKT, MCT1 and MCT4 were purchased from Santa Cruz Biotechnology (Santa Cruz, CA, USA). Anti-mouse and anti-rabbit IgG peroxidase conjugated antibodies and chemical reagents were from Sigma-Aldrich (St Louis, MO, USA). ECL, nitrocellulose membranes and protein assay kit were from Bio-Rad (Hercules, CA, USA). Culture media, sera, antibiotics and LipofectAMINE transfection reagent were from Invitrogen (Carlsbad, CA, USA). The ERβ selective agonist KB9520 was designed and synthesized by Karo Bio (Huddinge, Sweden). (KB9520 has been described previously [32, 45, 46]. The compound can be obtained following contact with Karo Bio AB [stefan.nilsson@karobio.se] and after signing of a Material Transfer Agreement together with a detailed protocol of planned study. A fee covering the cost of compound synthesis will be charged).

### Cell cultures and transfection

The biphasic MSTO-211H cell line was obtained from the Istituto Scientifico Tumori (IST) Cell-bank, Genoa, Italy; the epithelioid REN cell line was isolated, characterized and kindly provided by Dr. Albelda S.M. (University of Pennsylvania, Philadelphia; PA, USA). Cells were grown in standard conditions in RPMI medium supplemented with 10% FBS, 100 μg/ml streptomycin and 10 μg/ml penicillin at 37°C in a humidified environment containing 5% CO_2_. Cell cultures under hypoxic conditions were performed in 1% O_2_ gas mixture using a modulator incubator chamber. Mycoplasma infection was excluded by the use of Mycoplasma PlusTM PCR Primer Set kit from Stratagene (La Jolla, CA, USA). Cells grown to 80% confluence in tissue culture dishes were transiently transfected with the pcDNA3 AKT1-HA plasmid (Addgene, Cambridge, MA, USA) using LipofectAMINE reagent as described by the manufacturer. Gene silencing was achieved by specific siRNAs from Qiagen (Hilden, Germany).

### Proliferation assays

Cells were seeded at a density of 10 × 104 cells/well in 6-well plates in RPMI medium supplemented with 10% FBS, 100 μg/ml streptomycin and 10 μg/ml penicillin and incubated over-night at 37°C in a humidified environment containing 5% CO_2_ to allow adherence. Following treatment cells were trypsinized and stained with Trypan blue. The number of cells considered viable (unstained cells) was counted in a Bürker haemocytometer within 5 minutes after staining.

### Detection of intracellular lactate content

The intracellular lactate amount was analyzed using the Lactate Assay Kit from Sigma Aldrich (St Louis, MO, USA). Cells were trypsinized, washed with PBS and homogenized in 4 volumes of the Lactate Assay Buffer. Samples were centrifuged at 13.000 g for 10 minutes to remove insoluble material, and then stored at −80°C to inhibit lactate dehydrogenases activity. Lactate concentration was determined by an enzymatic assay, which results in a colorimetric (absorbance at 570 nm) product, proportional to the lactate present. For each assay performed, a standard curve was generated using lactate standards provided in the kit. Lactate concentration of samples was determined by plotting samples on the standard curve. Intracellular lactate was normalized to protein concentration of the sample.

### Cell lysis, immunoprecipitation and immunoblot

Cells were extracted with 1% NP-40 lysis buffer (1% NP-40, 150 mM NaCl, 50 mM Tris-HCl pH 8.5 mM EDTA, 10 mM NaF, 10 mM Na4P2O7, 0.4 mM Na3VO4) with freshly added protease inhibitors (10 μg/ml leupeptin, 4 μg/ml pepstatin and 0.1 Unit/ml aprotinin). Lysates were centrifuged at 13.000 × g for 10 minutes at 4°C and the supernatants were collected and assayed for protein concentration with the Bio-Rad protein assay method. For immunoprecipitation experiments, 2 mg of extracted protein for each treatment were incubated with specific antibodies for 1 hour at 4°C and 50 μl protein A-Sepharose beads. Proteins were separated by SDS-PAGE under reducing conditions. Following SDS-PAGE, proteins were transferred to nitrocellulose, reacted with specific antibodies and then detected with peroxidase-conjugate secondary antibodies and chemioluminescent ECL reagent. Densitometric analysis was performed using the GS 250 Molecular Image (Bio-Rad).

### RNA, DNA isolation and quantitative real-time PCR

Total RNA was extracted using the guanidinium thyocianate method. Starting from equal amounts of RNA, cDNA used as template for amplification in the real-time PCR (5 μg), was synthesized by the reverse transcription reaction using RevertAid Minus First Strand cDNA Synthesis Kit from Fermentas-Thermo Scientific (Burlington, ON, Canada), using random hexamers as primers, according to the manufacturer's instructions. The primers sequences are reported in Supplementary Figure S4. The real-time reverse transcription-PCR (RT-PCR) was performed using the double- stranded DNA-binding dye SYBR Green PCR Master Mix (Fermentas-Thermo Scientific) on an ABI GeneAmp 7000 Sequence Detection System machine, as described by the manufacturer. The instrument, for each gene tested, obtained graphical Cycle threshold (Ct) values automatically. Triplicate reactions were performed for each marker and the melting curves were constructed using Dissociation Curves Software (Applied Biosystems, Foster City, CA, USA), to ensure that only a single product was amplified.

### Multicellular spheroids

Multicellular spheroids were generated in non-adsorbent round-bottomed 96-well plates, as described [47]. The 96-well plates were coated with a 1:24 dilution of polyHEMA (120 mg/ml) (Sigma-Aldrich, St. Louis, MO) in 95% ethanol and dried at 37°C for 24 h. Before use, plates were sterilized by UV light for 30 min. For generation of multicellular spheroids, 103 cells were added into each well of polyHEMA-coated 96-well plate in medium added with 2.5% Matrigel and placed in a 37°C humidified incubator with 5% CO_2_. After an initiation interval of 24 hours, 50% of supernatant was replaced with fresh medium ± KB9520 (at final concentration of 10 nM) every 24 hours.

### *In vivo* experiments

#### Animals

CD1 nude mice (males, 6 weeks old; Charles River, Calco, Italy) received intra- peritoneal (i.p.) injections of 1 × 106 luciferase transduced MSTO-211H cells in 0.5 mL of RPMI medium. After anesthetization and i.p. injections of 0.3 mL of 15 mg/mL D-luciferin, tumor dimension and localization of luminescent cells was monitored using the *In Vivo* Imaging System (IVIS^®^) system 100 series (Xenogen Corporation, Hopkinton, MA, USA). Regions of interest were identified around the tumor sites and were quantified as total photon counts using Living Image software (Xenogen Corporation). To evaluate treatment toxicity, mice were weighed at the start and end of treatments. Mice were killed and necropsied after 25 days of treatment. *In vivo* experiments were approved by Istituto Scientifico Tumori (Genoa, Italy) ethical committee and conform to the relevant regulatory standards. Mice were maintained and handled under aseptic conditions, and were allowed access to food and water ad libitum.

#### Drug administration

An elapse of 15 days was allowed for the formation of detectable tumor nodules, assessed by IVIS^®^ imaging. Mice were then weighed and stratified into treatment groups of ten animals. Treatment protocols were done from the 15th day to the 40th day, and mice were analyzed every 4–5 days by IVIS^®^ imaging to assess tumor growth. One dose of KB9520 was used (10 mg/kg/day). KB9520 was dissolved in the vehicle (5% DMSO/40% PEG 400/55% water) and administrated once daily (days 15–40) by sub-cutaneous administration. Untreated animals were dosed with empty vehicle. At day 40 mice from the two groups were euthanized and necropsied. Tumors growing in the peritoneum were excised, and one part of the tumor tissues was immediately frozen and stored at −80°C for subsequent analysis.

### Statistical analysis

Statistical evaluation of the differential analysis was performed by one way ANOVA and Student's *t*-test. The threshold for statistical significance was set at *p* < 0.05. The statistical analysis of *in vivo* experiments was done by using R [48].

## SUPPLEMENTARY FIGURES


