# Supplementary material for: Intracellular lactate-mediated induction of estrogen receptor beta (ERβ) in biphasic malignant pleural mesothelioma cells
Source: Oncotarget. 2015 Jul 9;6(28):25121–34. doi: 10.18632/oncotarget.4486 (PMC4694819; doi:10.18632/oncotarget.4486)
Supplement: Supplementary file 1 [file oncotarget-06-25121-s001.pdf]

SUPPLEMENTARY FIGURES

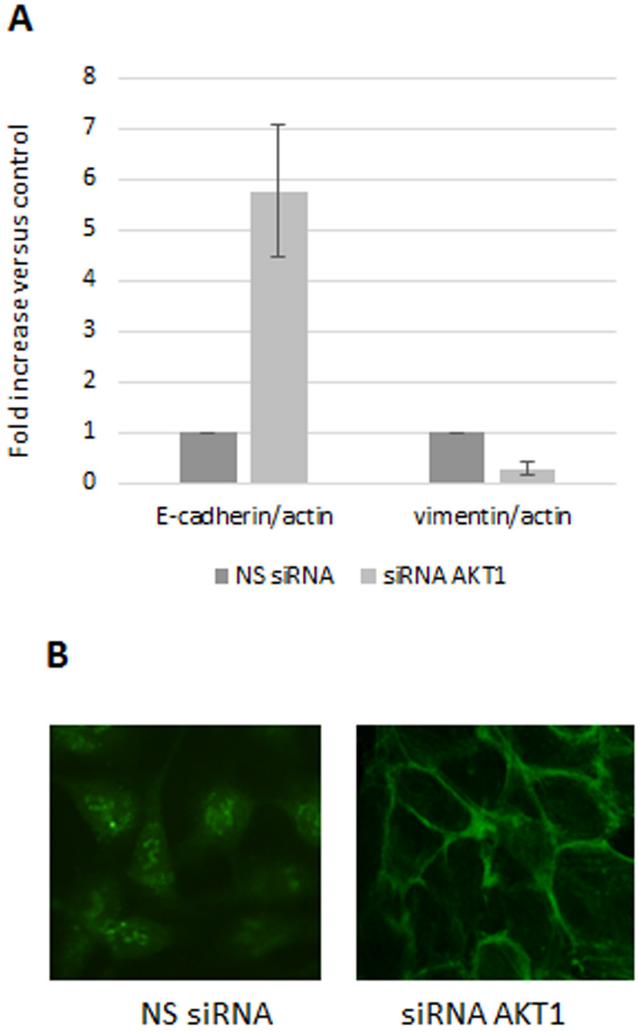

Supplementary Figure S1: E-Cadherin expression is significantly up-regulated in AKT1 silenced MSTO-211H cells. **A.** E-Cadherin and Vimentin mRNA expression evaluated by real-time PCR in control (NS siRNA) or AKT1-silenced (siRNA AKT1) MSTO-211H cells. Graph is representative of three independent experiments. Each bar represents mean ± s.d. $*p \leq 0.05$. β-actin mRNA was used as housekeeping gene. **B.** Representative immunofluorescence analysis of E-Cadherin expression in control (NS siRNA) or AKT1-silenced (siRNA AKT1) MSTO-211H cells.

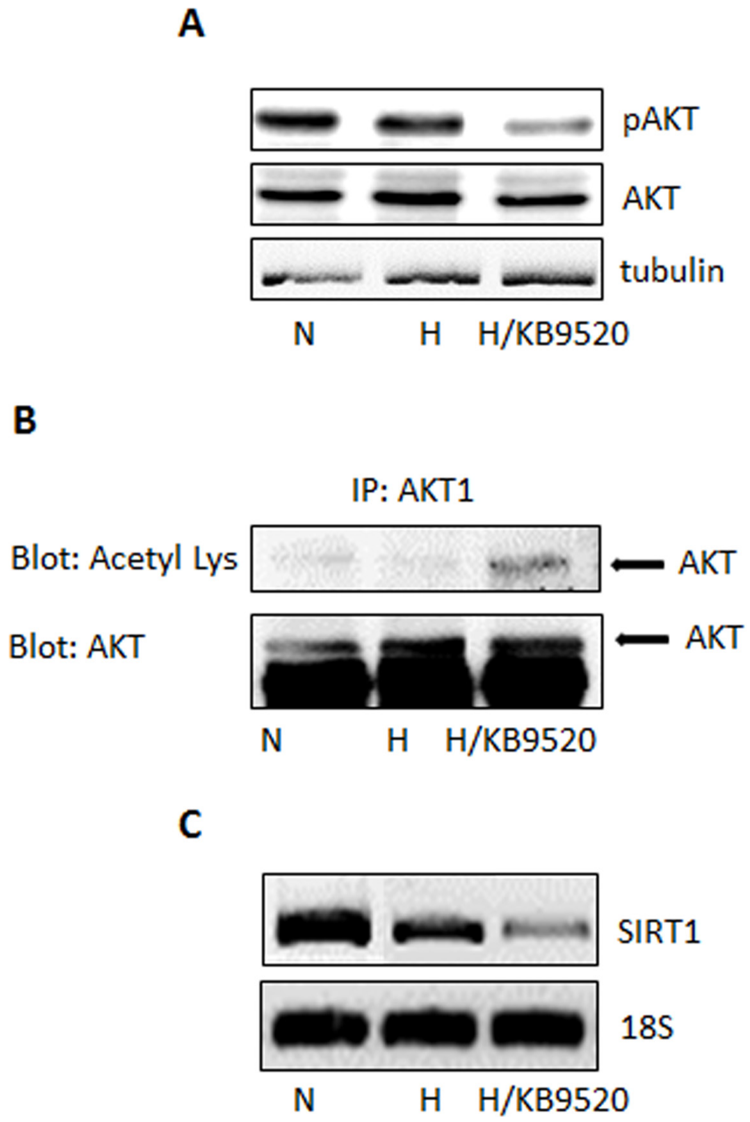

Supplementary Figure S2: AKT post-translational modifications and SIRT1 downmodulation in MSTO-211H cells treated with KB9520 in hypoxic conditions. A. Western blot analysis of phosphorylated and total AKT in MSTO-211H cells cultured 48 hours in normoxia (N) or hypoxia (H) ± 10 nM KB9520. Tubulin is included as a loading control. **B.** Immunoprecipitation analysis of acetylated AKT1 in MSTO-211H cells cultured 48 hours in normoxia (N) or hypoxia (H) ± 10 nM KB9520. **C.** RT-PCR analysis of *SIRT1* expressed by MSTO-211H cells in the culture conditions as described above. 18S rRNA was used as housekeeping gene.

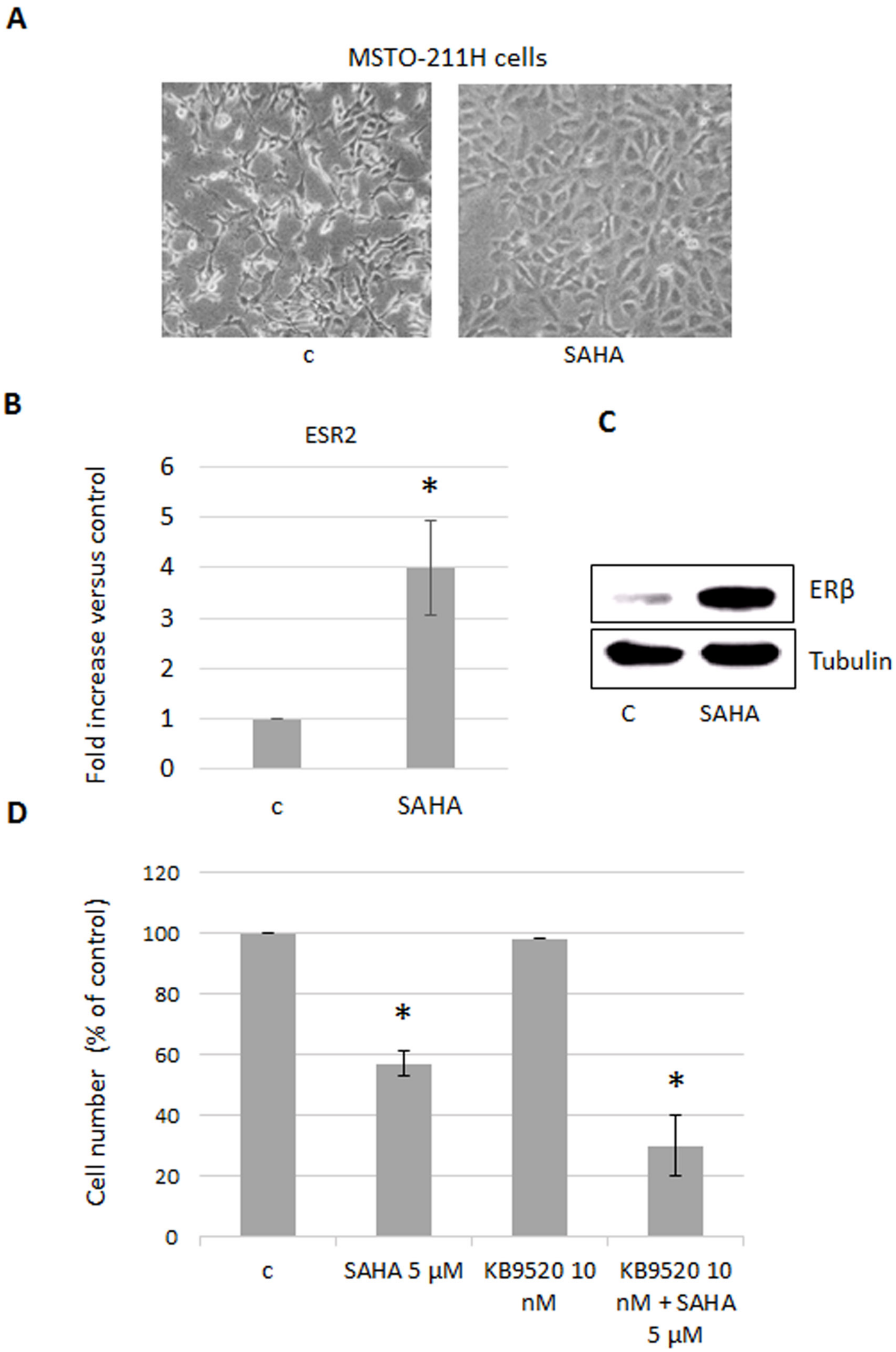

Supplementary Figure S3: SAHA treatment induces the acquisition of an epithelioid-like phenotype, ERβ expression and sensitivity to KB9520 in MSTO-211H cells. A. Phase contrast images (200× magnification) of MSTO-211H cells treated ± 5 μM SAHA for 72 hours. **B.** ERβ mRNA expression levels evaluated by real-time PCR and **C.** Western blot analyses in MSTO-211H cells treated ± 5 μM SAHA for 72 hours. 18S rRNA and tubulin, respectively, were used as control. **D.** Percentage of growth inhibition of MSTO-211H cells treated with 5 μM SAHA for 72 hours ± 10 nM KB9520 in the last 24 hours. Each graph is representative of three independent experiments. Each bar represents mean ± s.d. $*p \leq 0.05$.

RT PCR		
GENE	PRIMER FORWARD	PRIMER REVERSE
18S	5'-AAA CGG CTA CCA CAT CCA AG-3'	5'-CCT CCA ATG GAT CCT CGT TA-3'
AKT1	5'- GCT GGA CGA TAG CTT GGA-3'	5'-GAT GAC AGA TAG CTG GTG-3'
CDH1	5'-TGG GCT GGA CCG AGA GAG TT-3'	5'-ATC TCC AGC CAG TTG GCA GT-3'
SLC16A1	5'-TTT CTT TGC GGC TTC CGT TGT TG-3'	5'-TCA ATT TAC CCT TCA GCC CCA TGG-3'
SLC16A3	5'-TTT TGC TGC TGG GCA ACT TCT TCT G-3'	5'-TCA CGT TGT CTC GAA GCA TGG GTT T-3'
CD147	5'-AGC GGT TGG AGG TTG TAG G-3'	5'-TGG CAC GGA CTC TGA CTT G-3'
SIRT1	5'-CTG GAC AAT TCC AGC CAT CT-3'	5'-GGG TGG CAA CTC TGA CAA AT-3'
EPAS1	5'-GGG GAT CAG CGC ACA GAG TT-3'	5'-TGG GCT GAC GAC AGG CTG TA-3'

REAL TIME PCR		
GENE	PRIMER FORWARD	PRIMER REVERSE
18S	5'-CCC ACT CGG CAC CTT ACG-3'	5'-TTT CAG CCT TGC GAC CAT ACT-3'
ESR2	5'-GTC AGG CAT GCG AGT AAC AA-3'	5'-GGG AGC CCT CTT TGC TTT TA-3'

Supplementary Figure S4: List and sequence of the primers used for RT-PCR and Real Time PCR analyses.
